# Simultaneous Identification of Both *MFSD8* and *RDH12* Pathogenic Variants in a Chinese Family Affected With Retinitis Pigmentosa

**DOI:** 10.3389/fgene.2021.715100

**Published:** 2021-09-09

**Authors:** Yihui Wang, Yanling Teng, Desheng Liang, Zhuo Li, Lingqian Wu

**Affiliations:** ^1^Center for Medical Genetics, Hunan Key Laboratory of Medical Genetics & Hunan Key Laboratory of Animal Models for Human Diseases, School of Life Sciences, Central South University, Changsha, China; ^2^Hunan Jiahui Genetics Hospital, Changsha, China

**Keywords:** retinitis pigmentosa, *MFSD8* gene, *RDH12* gene, molecular testing, intron retention, exon skipping

## Abstract

Retinitis pigmentosa (RP) is characterized by tremendous genetic and phenotypic heterogeneity. Here, we investigate the pathogeny of RP in a family to provide evidence for genetic and reproductive counseling for families. Although this pregnant woman of 8^+3^ weeks presented with RP, her first baby was born with RP, epilepsy, and cerebellar atrophy. The research identified a compound heterozygous mutation (c.998+3_998+6del/deletion) in the *MFSD8* gene of the first born, explaining the cause of the proband’s disease, which cannot explain the mother’s. Then, a homozygous mutation c.343+1G > A in *RDH12* of the mother was found. RT-PCR is employed to find that there is a skipping of exon 10 in *MFSD8* and a 15-nucleotide retention of intron5 in *RDH12*. The coexistence of two independent instances of RP caused by distinct genes in one pedigree is demonstrated. Based on the diagnosis, a prenatal diagnosis performed on the fetus found that the fetus’s *MFSD8* is affected by the same mutation as the proband. The research underscoring the complexity of RP and the need for the combination of extensive molecular genetic testing and clinical characterization in addition expands the spectrum of *MFSD8* mutations. Finally, it is expected that the family members would be prevented from reproducing children with the similar disease.

## Introduction

Retinitis pigmentosa (RP) is the most common type of inherited retinal dystrophy with a prevalence of approximately 1/4000 (Hamel, 2006). RP is generally progressive, characterized by the initial degeneration of rod photoreceptors followed by the loss of cone photoreceptors, which typically manifests with night blindness in adolescence and concentric visual field loss, and later in life, central vision loss occurs because of cone dysfunction (Salmaninejad et al., 2019). Bone spicule pigmentation, a waxy pallor of the optic nerve, and attenuation of retinal vessels bear the hallmarks of RP, but in the early stages, a fundus examination may appear normal (Verbakel et al., 2018).

The genetic and clinical heterogeneity of RP is relatively high. Clinically, it is divided into nonsyndromic RP (NSRP) and syndromic RP, and the majority of RP cases are nonsyndromic. Mutations in more than 80 genes are associated with NSRP, explaining 60–70% of cases (Solaguren-Beascoa et al., 2020). Some of the related genes can cause various clinical visual phenotypes in different individuals even in the same family (Schuster et al., 2005). Just as the clinical presentations related to *RDH12* mutations include Leber congenital amaurosis (LCA), early onset severe retinal dystrophy, autosomal recessive RP (ARRP), autosomal dominant RP, and cone-rod dystrophy (Zou et al., 2019). *RDH12* encodes retinol dehydrogenase 12, an enzyme expressed in photoreceptors that reduces all-trans-retinal to all-trans-retinol (Haeseleer et al., 2002). *RDH12* protects internal fragments from retinal-induced cytotoxicity by reducing free all-trans retina (Haeseleer et al., 2002; Chen et al., 2012; Hofmann et al., 2016).

Not only can RP develop alone, but it also results as a complication of many complex syndromes, manifested as syndromic RP, such as neuronal ceroid lipofuscinoses (NCLs), which is characterized by progressive motor and mental regression in combination with seizures, ataxia, and visual impairment (Zare-Abdollahi et al., 2019). The visual impairment includes a broad range of diseases from localized macular to widespread retinal degeneration, such as RP (Magliyah et al., 2019). NCL type 7 (CLN7) is one subtype of NCL, caused by the mutations in the major facilitator superfamily domain containing the Protein-8 gene (*MFSD8*). *MFSD8* encodes a lysosomal membrane protein, which could transport small solutes by electrochemical gradients.

Due to the complex, overlapping phenotypes, for some rare families, although different patients mainly suffered from visual impairment, they are finally diagnosed with more than one independent genetic cause (Birtel et al., 2020). Therefore, it is essential for the individual medical management to identify distinct mutations and inheritance modes in these family members. With the development of genetic technologies, next-generation sequencing is combined with other molecular genetic testing technologies and have made it more feasible to achieve extensive diagnoses.

In this study, we describe a family with three patients displaying complex manifestations, in which the proband presented with typical syndromic phenotypes of CLN7 resulting from two novel *MFSD8* mutations, and another two affected individuals presented with NSRP caused by *RDH12* mutations on both alleles. To our knowledge, this is the first report that both rare diseases of *RDH12*-associated RP and CLN7 coexist in one family. Furthermore, based on the genetic testing results, a fetus in this family was prenatally diagnosed with CLN7.

## Materials and Methods

### Patients

**FIGURE 1:**
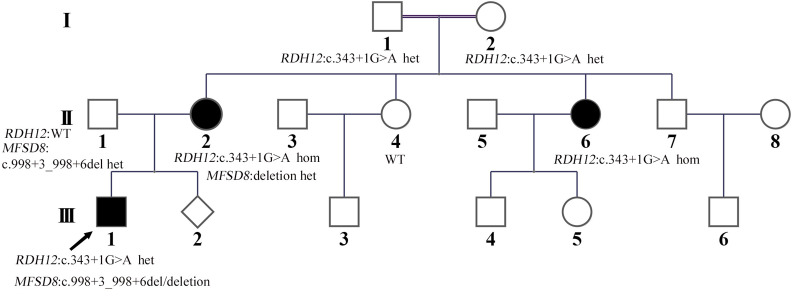
Familial pedigree. The proband (III:1) is marked with an arrow. His disease courses started from visual loss at about three and a half years old. The mother of the proband (II:2) was from a consanguineous family; her and her older sister (II:6) developed symptoms of night blindness in the third decade of life, and the mother of the proband’s (II:2) condition of the fundus was the same as that of the proband. Only for the proband, seizures were observed as the second symptom after decreased vision from about 4 years old.

A Chinese family suffering from vision problems and/or epilepsy was recruited from the Hunan Jiahui Genetic Hospital, Changsha, China, including a total of 16 members (Figure 1). All three affected patients had received a diagnosis of RP.

This study fully complies with the tenets of the Declaration of Helsinki, and it is approved by the Ethics Committee of Central South University (No. 2016102101). The patient’s parents have signed written informed consent.

### Mutation Analysis

The genomic DNA (gDNA) of the related family members was extracted from their blood leukomonocyte or amniotic fluid by standard methods according to the manufacturer (FUJIFILM, Tokyo, Japan). First, 50 ng DNA was interrupted to around 200 bp by fragmentation enzymes. Second, the DNA fragments were ligated with barcoded sequencing adaptors, and fragments about 320 bp were collected by XP beads. The Novaseq6000 platform (Illumina, San Diego, United States) was used for sequencing the genomic DNA of the family. Finally, raw image files were processed using CASAVA v1.82 for base calling and generating raw data.

A comprehensive tool called Sprinkle was used for CNV calling. It included the XHMM (Fromer and Purcell, 2014) PCA method to remove sequencing noise and the CNVKit (Talevich et al., 2016) fix module to perform GC and bias correction, and then copy number calculation and CNV identification were performed in exons and long segment areas. The results of CNVs were identified and mapped by referring to the hg19 version of the human genome and publicly available database. Finally, the map was drawn to indicate the relationship between copy number (*Y*-axis) and exons (*X*-axis).

### Mutation Validation

The detected CNVs were corroborated by qPCR with the Maxima SYBR Green qPCR Master Mix (2X) (Thermo, United States) on an ASA-9600 real-time PCR cycler (BaiYuan Gene-Tech, Suzhou, China). The copy number of gDNA was measured as the ratio of the ^ΔΔ^CT after normalization with ALB (Shi et al., 2020). Statistical analysis was performed using Prism GraphPad 5. The identified variants were confirmed by Sanger sequencing. It was also employed to determine whether the variant cosegregated with the disease phenotype.

### Identification of Splice Transcripts

To analyze the splicing mutation, total RNA from blood leukocytes of the proband and controls was extracted using a standard Trizol method. cDNAs were synthesized from RNA using the RevertAid First Strand cDNA Synthesis Kit (Thermo, United States). The PCR primers were designed using Primer5 software (Premier Biosoft International, Palo Alto, CA, United States). The details of all the PCR primers are listed in Supplementary Table 1.

## Results

### Clinical Phenotype

**FIGURE 2:**
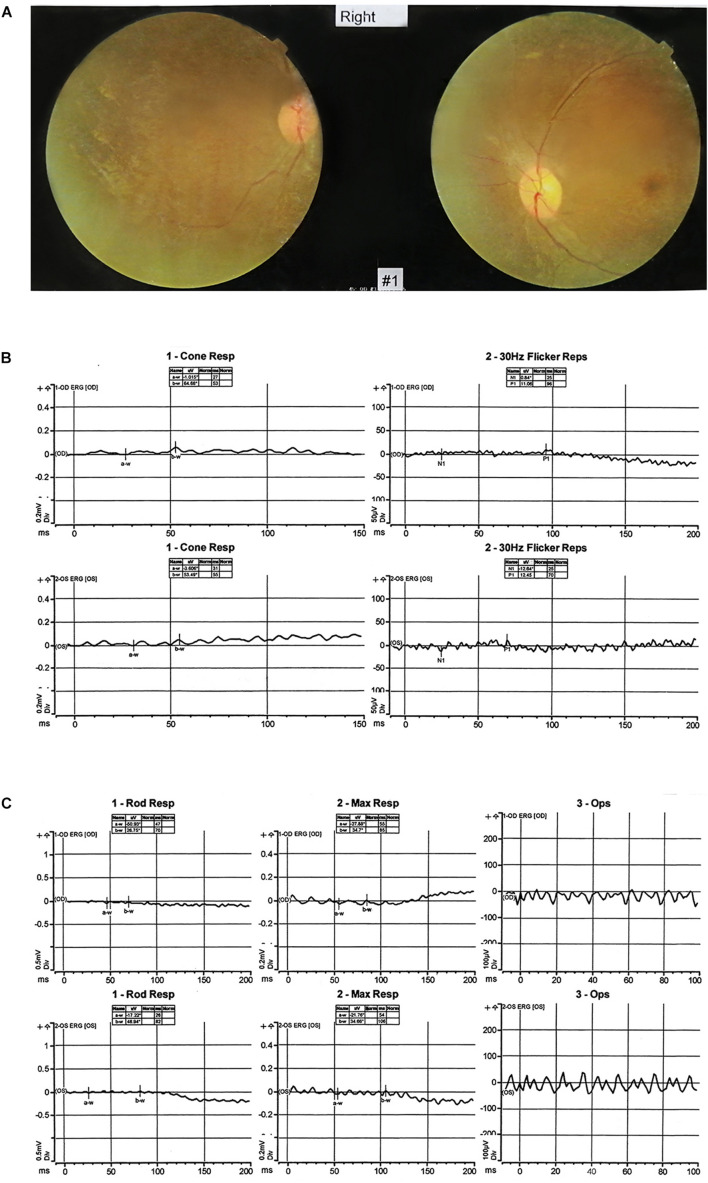
Clinical features. **(A)** The fundus imaging result of the proband (III:1). The fundus imaging shows that the eye position is not correct, the retinal artery is thinned, and the blood vessel is accompanied by a white sheath. **(B,C)** The electroretinography result of the proband (III:1). After dark adaptation for 20 min, the rod cell response b-w wave basically disappeared, the maximum mixed response a-w wave basically disappeared, and the peak amplitude of oscillatory potentials decreased. After light adaptation for 10 min, the peak amplitude of the cone response b-w wave decreased significantly, and the 30-Hz scintillation light response amplitude decreased. It reflects that the inner and outer layers of the retina are damaged, and the retina is in an ischemic state.

The proband (III:1) whom the research investigated is a 10-year-old boy from a nonconsanguineous family in China (Figure 1). The proband’s vision began to decline gradually at the age of three and a half. By the age of 4, he had developed whole body seizures when sleeping at night. The duration ranged from a few seconds to 1 min or so. The symptom, which could be relieved naturally, occurred once in about 1–2 weeks with twitches occasionally happening during high fever. There was no regularity of seizures or an obvious cause. After 2 years, he was diagnosed with severely declined vision at Hunan Children’s Hospital: macular dysplasia and RP (Figure 2). About a year later, he had a head MRI at a local hospital because of a seizure, and it indicated atrophy of the cerebellum, deepened sulci of the cerebral hemispheres, and enlarged adenoids. An EEG showed no abnormality in background activity, abnormal discharge in the left parietal, and left middle temporal epilepsy. He was treated with sodium valproate tablets. The child’s symptoms gradually improved. The seizures occurred once or twice a month, and the duration was shorter.

The mother of the proband (II:2) gradually found her vision occasionally blurred and decreased, especially at night, after giving birth at the age of 21. It was suggested by her doctor that the condition of the fundus of the pregnant woman was the same as that of the child, considering the possibility of RP.

As for siblings, II:2 has an elder sister (II:6) whose vision decreased at about 25, especially at night. She has given birth to two healthy children of both genders. There are two other siblings, II:4 and II:7, both of whom, nevertheless, have normal vision.

### Genotype of the Patients

**FIGURE 3:**
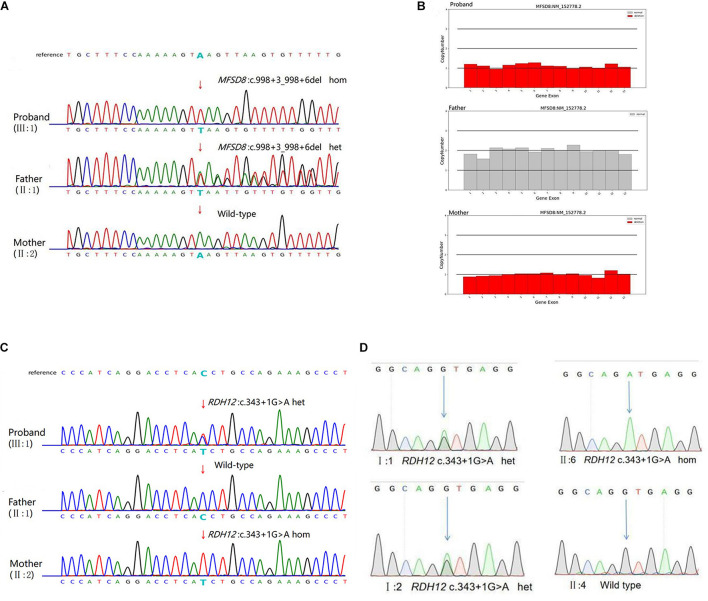
Genetic characterization of the family. **(A)** The candidate causal mutation c.998+3_998+6del of *MFSD8* discovered via WES was confirmed by Sanger sequencing. A homozygous deletion (c.998+3_998+6del) in *MFSD8* was found in the proband and was not detected in his mother. A heterozygous mutation was found in his father. The red arrow indicates the site of the mutation. **(B)** *MFSD8* detected by WES-CNV in the family. WES-CNV reveals an obvious deletion in the proband inherited from his mother. There was nothing abnormal in the same position of his father. **(C)** The mutation c.343+1G > A of *RDH12* was confirmed by Sanger sequencing. A heterozygous mutation (c.343+1G > A) in *RDH12* was found in the proband. His father did not show this mutation. A homozygous mutation (c.343+1G > A) in *RDH12* was found in his mother. The red arrow indicates the site of the mutation. **(D)** Cosegregation analyses among the consanguineous family. Both parents (I:1 and I:2) were heterozygous for c.343+1G > A of *RDH12*. The sister (II:6) with a similar phenotype to II:2 had the same homozygous mutations, and another sister (II:4) with a normal phenotype was wild type. The blue arrow indicates the site of the mutation.

Next-generation sequencing was carried out in the exome of the proband. The proband was identified with a homozygous c.998+3_998+6del of *MFSD8* that has not been reported before, and it was confirmed by Sanger sequencing (Table 1 and Figure 3A). Cosegregation analyses among the family was also conducted, and his father suffered a heterozygous mutation, but his mother did not.

**TABLE 1:** The result of WES.

Gene	Mutation location	HGVS	Results	ACMG classification	Diseases and inheritance
*MFSD8*	intron10 chr4:127930677-127930680	intron variant NM_152778.3:c.998 +3_998+6del	III:1 hom II:1 het II:2 WT	VUS* (PM2+PM3+PP3)	Neuronal ceroid lipofuscinoses 7, AR
*RDH12*	intron5 chr14:67725255-67725255	splicing NM_152443.3: c.343+1G + A	III:1 het II:1 WT II:2 hom	Pathogenic (PVS1+PM2+PP1)	ARRP, AR

*After the follow-up experiments, c.998+3_998+6del in the *MFSD8* gene is classified “Pathogenic (PVS1+PM2+PM3+PP1)”.

Therefore, it is doubted whether the mother had a loss of heterozygosity. To further explore the source of variation of the proband, a WES-CNV analysis of the core family was done, and the results confirmed that our suspicion was correct (Figure 3B). The c.998+3_998+6del of *MFSD8* gene is a compound heterozygous mutation (c.998+3_998+6del/deletion) and is not homozygous (Table 2).

**TABLE 2:** The result of WES-CNV.

Case	Chromosome location	Chromosome segment	CNV type (Deletion/duplication)	size	Classification	Segment corresponding disease	Clinically relevant genes
Proband (III:1)	chr4:127920530-127965957	4q28.2	Deletion	45.43Kb	Pathogenic (PVS1_Stand-alone: Full gene deletion)	Neuronal ceroid lipofuscinoses 7, Macular dystrophy with central cone involvement	*MFSD8*

The mutation of *MFSD8* (OMIM:611124) can lead to the autosomal recessive genetic disease neuronal ceroid lipofuscinoses 7 (OMIM:610951). With a WES-CNV test performed on the samples, it was found that the 45.43Kb region of 4q28.2 on chromosome 4 was deleted, covering exons 1–13 of the *MFSD8* gene. The WES-CNV is not reported in DGV, DECIPHER, OMIM, UCSC, and PubMed public databases. WES-CNV results showed that the proband’s 1–13 exons heterozygous deletion of MFSD8 were from the mother.

According to ACMG guidelines, mutation c.998+3_998+6del of *MFSD8* gene is interpreted as a variant of uncertain significance (VUS) because this mutation with extremely low frequency (PM2) might affect splicing predicted by splice AI (PP3). Mutation c.998+3_998+6del is not a variation in canonical splice sites and cannot be simply classified as a null variant. Therefore, only the CNV can be classified as “pathogenic” (PVS1_Stand-alone: full gene deletion) (Brandt et al., 2020) for this compound heterozygous mutation (c.998+3_998+6del/deletion) in the *MFSD8* gene. The follow-up experiments were necessary to achieve the classification of “likely pathogenic” because the CLN7 disease was a recessive genetic disease (PM3). Because this mutation was located near the splicing site, abnormal cleavage of the *MFSD8* gene transcript possibly could result.

Later, considering that the phenotype of the mother also suffered decreased vision, which was not shown as NSRP, and that the c.998+3_998+6del of *MFSD8* did not conform to autosomal recessive inheritance, the WES result was further screened, and a homozygous variant, c.343+1G > A of *RDH12*, was found. The candidate causal genes discovered via WES were then confirmed by Sanger sequencing (Figure 3C), and cosegregation analyses among the consanguineous family were also conducted, indicating that the parents (I:1 and I:2) of the mother of proband (II:2) are the carriers, respectively. The other patient (II:6) also carries the same mutation as the II:2., but II:4, another female member in the family with a normal phenotype, are wild type. All results are shown in Figure 3D. Mutation c.343+1G > A of *RDH12* can be classified as pathogenic because it is a variant of 1–2 amino acids near the splicing site (PVS1) and absent from controls (PM2), cosegregated in multiple affected family members (PP1).

### The Transcript Consequence Was Ascertained

**FIGURE 4:**
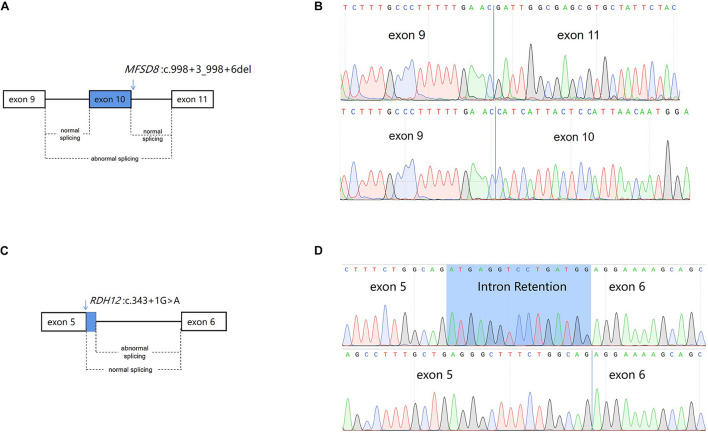
Schematic diagram of alternative splicing isoforms. **(A)** Schematic presentation of *MFSD8* splicing. The arrows above the diagram indicate the positions of the mutation c.998+3_998+6del of MFSD8. **(B)** Sanger sequencing analysis of the proband (III:1) showed exon skipping in the cDNA. **(C)** Schematic presentation of *RDH12* splicing. The arrows above the diagram indicate the positions of the mutation c.343+1G > A of *RDH12*. **(D)** Sanger sequencing analysis of the mother (II:2) showed an intron retention in the cDNA.

To determine the impact of the described variants on splicing, RNA from lymphocytes of the proband and his mother were extracted and reversed into cDNA. Sequence analysis of the aberrant PCR product confirmed the skipping of exon 10 in the mutant transcript (PVS1), resulting in a direct connection between exons 9 and 11 in *MFSD8* (Figures 4A,B). This result verifies our previous conjecture. According to the result, the mutation can be interpreted as likely pathogenic.

The splicing abnormality caused by *RDH12* was different from the previous one. Sanger sequencing of RT-PCR products revealed that a c.343+1G > A variant disrupted the donor splice site (SS) of intron 5, which leads to activation of another downstream intronic cryptic SS. The activation of the cryptic SS causes a 15-nucleotide retention of intron 5 (Figures 4C,D), eventually resulting in the insertion of five amino acids (Supplementary Figure 1). To identify the mutated RDH12 proteins, either hydrophobicity or a net charge density SOSUI server was run, and outputs are analyzed (Supplementary Figure 2).

### Prenatal Diagnosis in This Family

Prenatal diagnosis was carried out after identification of the causative variant to CLN7 in the family. Maternal contamination was excluded in the DNA from the amniotic fluid, and then the genotype of the given location in the gene of *MFSD8* was tested.

**FIGURE 5:**
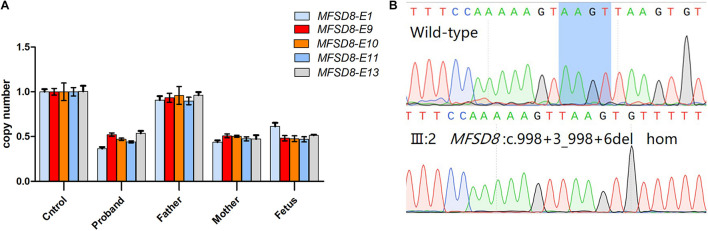
Prenatal genetic analysis of the fetus. **(A)** Quantitative real time PCR analysis of *MFSD8* exon 1, 13, 9–11 in the family. The control genome was normalized to one. Different colors show different exons: light blue for exon 1, red for exon 9, orange for exon 10, deep blue for exon 11, gray for exon 13. The *X*-axis shows different samples. **(B)** Sanger sequencing analysis of the fetus(III:2) showed intron variants in c.998+3_998+6del.

The fetus was found to be a compound heterozygous mutation (c.998+3_998+6del/deletion) in the *MFSD8* gene for the pathogenic variant. Among them, the qPCR results showed that exons 1, 13, 9–11 of the fetal *MFSD8* gene had a heterozygous deletion (Figure 5A). Sanger sequencing results showed that the *MFSD8* gene had a homozygous mutation of c.998+3_998+6del (Figure 5B). Therefore, the fetus was predicted to exhibit variant late-infantile NCLs. This is the first report of prenatal diagnosis for CLN7 disease.

## Discussion

We report a family with RP. The proband has more serious phenotypes, such as epilepsy and cerebellar atrophy. In this study, we identified the genetic pathogen of the patients, including the proband, his mother, and his aunt. The causes of their inherited retinal degeneration were two completely different genes. The proband carried a compound heterozygous mutation (c.998+3_998+6del/deletion) in the *MFSD8* gene, one from his father (c.998+3_998+6del), and the deletion was found to be from his mother by CNV testing, by which their entire *MFSD8* gene was deleted. It also explained why the proband had symptoms of epilepsy in addition to eye diseases because mutations in the *MFSD8* gene cloud cause CLN7, which mainly manifests as RP, language delay, seizures, etc. The RP of his mother and aunt was caused by the homozygous mutation of *RDH12* (c.343+1G > A), which originates from their parents, and their parents are heterozygous. The mutations did not appear in the two population databases of 1000 g and ExAC.

We discovered and reported a new pathogenic mutation (c.998+3_998+6del) of *MFSD8* for the first time and performed functional verification, confirming that this mutation can cause the exon 10 skipping and indeed affect RNA shearing and protein translation. Also, a novel deletion involving the whole *MFSD8* gene was identified by WES. The c.343+1G > A pathogenic variant of *RDH12* has been reported before (Zou et al., 2019), but it is shown for the first time that this mutation causes a 15-nucleotide retention of intron 5.

Mutations in *RDH12* are the underlying cause in 3–7% of retinal dystrophy cases (Zou et al., 2019). The most severe phenotype of *RDH12*-related recessive retinopathy is represented by LCA. Early onset RP and LCA have both clinical and genetic overlap, and both of these disorders represent the continuum of retinal dystrophies and are divided by indistinct criteria based on the age of onset. *RDH12* retinopathy is characterized by impaired visual function starting at 2–4 years, generally, which is inconsistent with the finding in our study. In this study, both patients from the consanguineous family developed night blindness and vision loss in the third decade. The possible reason is that this site is a canonical splice site, which only caused the insertion of five amino acids but did not cause the premature termination of protein translation. In addition, the insertion position was at 115–119, not located in a mutational hot spot or critical and well-established functional domain (e.g., active site of an enzyme).

The loss-of-function variants in the *MFSD8* gene are mainly observed in NCL 7 (CLN7), which presents with deterioration of cognitive and motor skills in combination with seizures, ataxia, and progressive loss of vision. Recently, one study showed that the *MFSD8* mutation can also cause nonsyndromic macular dystrophy with central cone involvement (CCMD). Actually, CLN7 and nonsyndromic CCMD are not two completely independent diseases; rather, they are allelic diseases. Some studies suggest that there is a genotype–phenotype model, a combination of a severe variant exactly resembling nonsense and frameshift and a mild variant such as missense in a compound heterozygous state that causes nonsyndromic CCMD, but two severe mutations cause CLN7 (Roosing et al., 2015). In fact, our results support the threshold model. In this study, our proband carries a compound heterozygous mutation (c.998+3_998+6del/deletion) in the *MFSD8* gene with one *MFSD8* gene completely deleted while the other chromosome exon 10 of *MFSD8* was skipped at the level of transcription. Both of them are relatively severe mutations. It seems logical to propose the threshold model by the residual activity of the *MFSD8* gene. *MFSD8* is mainly expressed in the brain and the eyes antenatally although it is expressed in almost all tissues ubiquitously at a relatively low level (Zare-Abdollahi et al., 2019). When *MFSD8* suffices for its proper function in all organs other than the eyes, nonsyndromic CCMD is the only phenotype. Certainly, there is the possibility that reasons for the phenotypic variation are nongenetic factors, such as lifestyle or environmental influence.

However, progression of the disease, severity of cone dystrophy, age of onset, and possibility of night blindness vary greatly among different affected individuals with cone–rod dystrophies. Retinopathy caused by *MFSD8* mutations is either not further specified or suggestive for RP. Therefore, it is difficult to distinguish the retinal dystrophy of patients with autosomal recessive mutation in the *MFSD* gene. There are reports of RP patients with a homozygous variant (c.1445G > C, p.Arg482Pro) in exon 13 in the *MFSD8* gene (Birtel et al., 2018).

In our study, the first prenatal diagnosis of NCL (CLN7) with *MFSD8* is reported. We carried out a prenatal diagnosis for this family based on the two identified pathogenic variants of the *MFSD8* gene. After excluding maternal contamination in the amniotic fluid, the fetus showed the same genotype as the proband. This result indicated that the fetus would probably have the same phenotype as the proband. Considering fetal viability, the mother conducted nondirective counseling on termination or continuation of the pregnancy. Finally, the mother made the decision to terminate the pregnancy after all available medical options were considered.

In conclusion, this rare case enriched our knowledge about the genotype/phenotype correlation with ARRP. Our finding extends the variant spectrum of the *MFSD8* gene and provides a solution to bearing healthy offspring for patients by prenatal diagnosis.

## Data Availability Statement

The datasets presented in this study can be found in online repositories. The names of the repository/repositories and accession number(s) can be found below: NCBI GenBank, accession numbers: MZ571209 and MZ571210.

## Ethics Statement

The studies involving human participants were reviewed and approved by This study was approved by an Ethics Committee of the Central South University (No.2016102101). Written informed consent to participate in this study was provided by the participants’ legal guardian/next of kin. Written informed consent was obtained from the individual(s), and minor(s)’ legal guardian/next of kin, for the publication of any potentially identifiable images or data included in this article.

## Author Contributions

YW: data curation, formal analysis, investigation, validation, writing – original draft, and writing – review and editing. YT: writing – review and editing. DL, ZL, and LW: funding acquisition, project administration, and writing – review and editing. All authors contributed to the article and approved the submitted version.

## Conflict of Interest

The authors declare that the research was conducted in the absence of any commercial or financial relationships that could be construed as a potential conflict of interest.

## Publisher’s Note

All claims expressed in this article are solely those of the authors and do not necessarily represent those of their affiliated organizations, or those of the publisher, the editors and the reviewers. Any product that may be evaluated in this article, or claim that may be made by its manufacturer, is not guaranteed or endorsed by the publisher.

## Supplementary Material

Supplementary Figure 1 Structure modeling of wild-type and mutated RDH12.

Supplementary Figure 2 Result of SOSUI prediction to mutated RDH12.

Supplementary Table 1 The primers for sanger sequencing.
