# Supplementary material for: Simultaneous Identification of Both MFSD8 and RDH12 Pathogenic Variants in a Chinese Family Affected With Retinitis Pigmentosa
Source: Front Genet. 2021 Sep 9;12:715100. doi: 10.3389/fgene.2021.715100 (PMC8458757; doi:10.3389/fgene.2021.715100)
Supplement: Supplementary Figure 1 — Structure modeling of wild-type and mutated RDH12. [file Table_1.DOCX]

Supplementary Material

Table S1

The primers for sanger sequencing

| Primer name | F, 5′ → 3′ | R, 5′ → 3′ |
| --- | --- | --- |
| *RDH12*-EXON3 | GACGGAGAGGAGCAGAGAAG | GCCCTGACTTTCTCCTCTGT |
| *RDH12*-EXON4 | CCACCTGCACCGATAAACAG | GGCCACAGTGTAAGTTGGTG |
| *RDH12*-EXON5 | ACTGTGAAAAGCCCGAAGTG | GCCCAGTCTATCTACCCACC |
| *RDH12*-EXON6 | CTGTTACAGGCAGCTAGGGG | CTCTATGAAGGCCCCTGGAC |
| *RDH12*-EXON7 | GGTCAAGGGCAGAGGTACAA | TGCATGTTTGACAGCCTGAC |
| *RDH12*-EXON8 | TGTTTCCTGAGTCCCTCCTTC | TCAGGCACAAACTCAGCTTC |
| *RDH12*-EXON9 | GAAACATTCTGAGAAAGGGACCA | CAGGTGTGCAAACATTCCCA |
| *RDH12*-RT | GCTGGTCACCTTGGGACTGCT | CTTCTCGCTCTGGAGGTCGTG |
| *MFSD8*-EXON10 | TGTATGCCTGGACTCAAGAAC | TACCAAAGAAATGCTGTAATGTA |
| *MFSD8*-Q-EXON9 | ATGAAGCTCAGGTTCCCCAA | GGTTAAAACATAATCTGATTCAGATGAA |
| *MFSD8*-Q-EXON10 | TGGACTCAAGAACAAGCTGTGTTAT | TTTCCTGGTATATCCATTTGCATTG |
| *MFSD8*-Q-EXON11 | ATTGGCGAGCGTGCTATTCTA | CCATTGCAGTGCATTACTTGTTG |
| *MFSD8*-RTR | ATTGACCAGGTTGCTGTTGTG | GGTATTAGGGATTGAATTATTGTGC |
| *MFSD8*-Q-EXON1 | GCTTGTTTTGCCAGCTTCACG | AGACTGAGGGGTCCCTCCACC |
| *MFSD8*-Q-EXON13 | CAATATCAATCTGCTTGTCCTCCAT | CAATATCAATCTGCTTGTCCTCCAT |


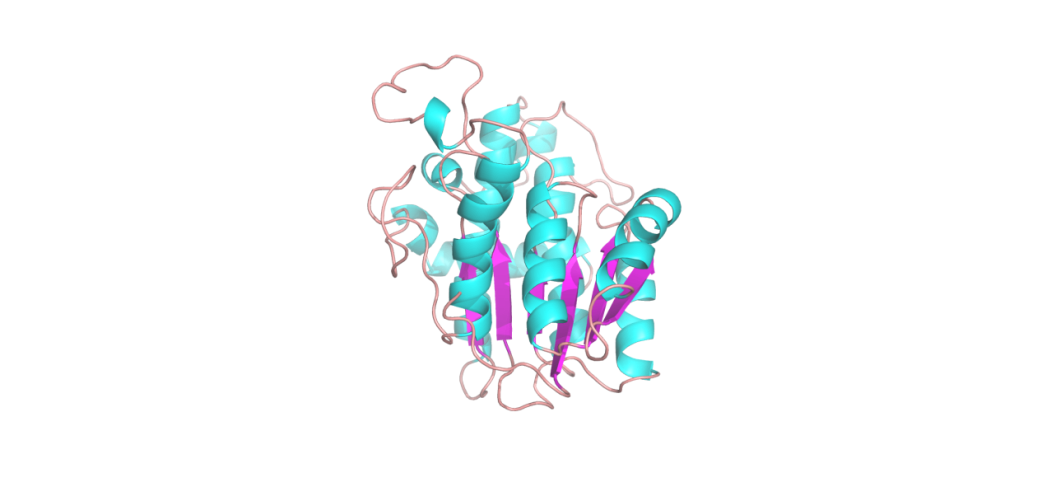

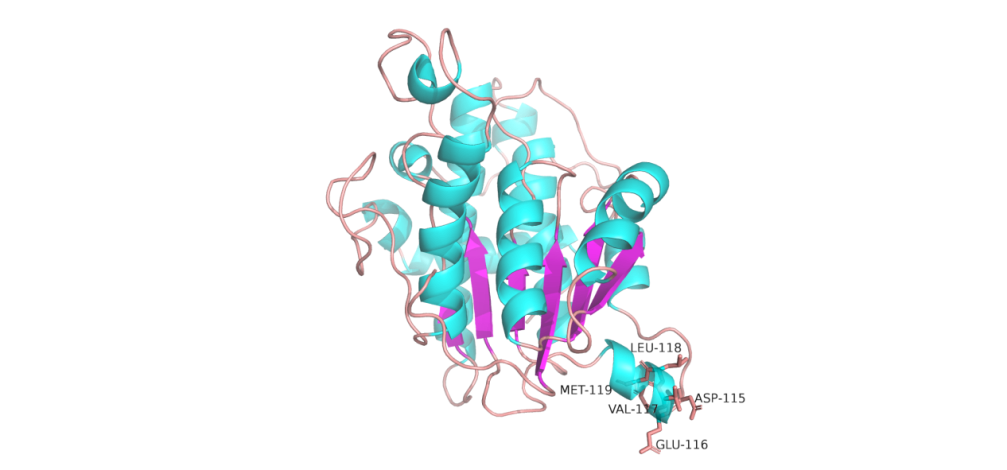


**Fig.S1 Structure modeling of wild-type and mutated RDH12**


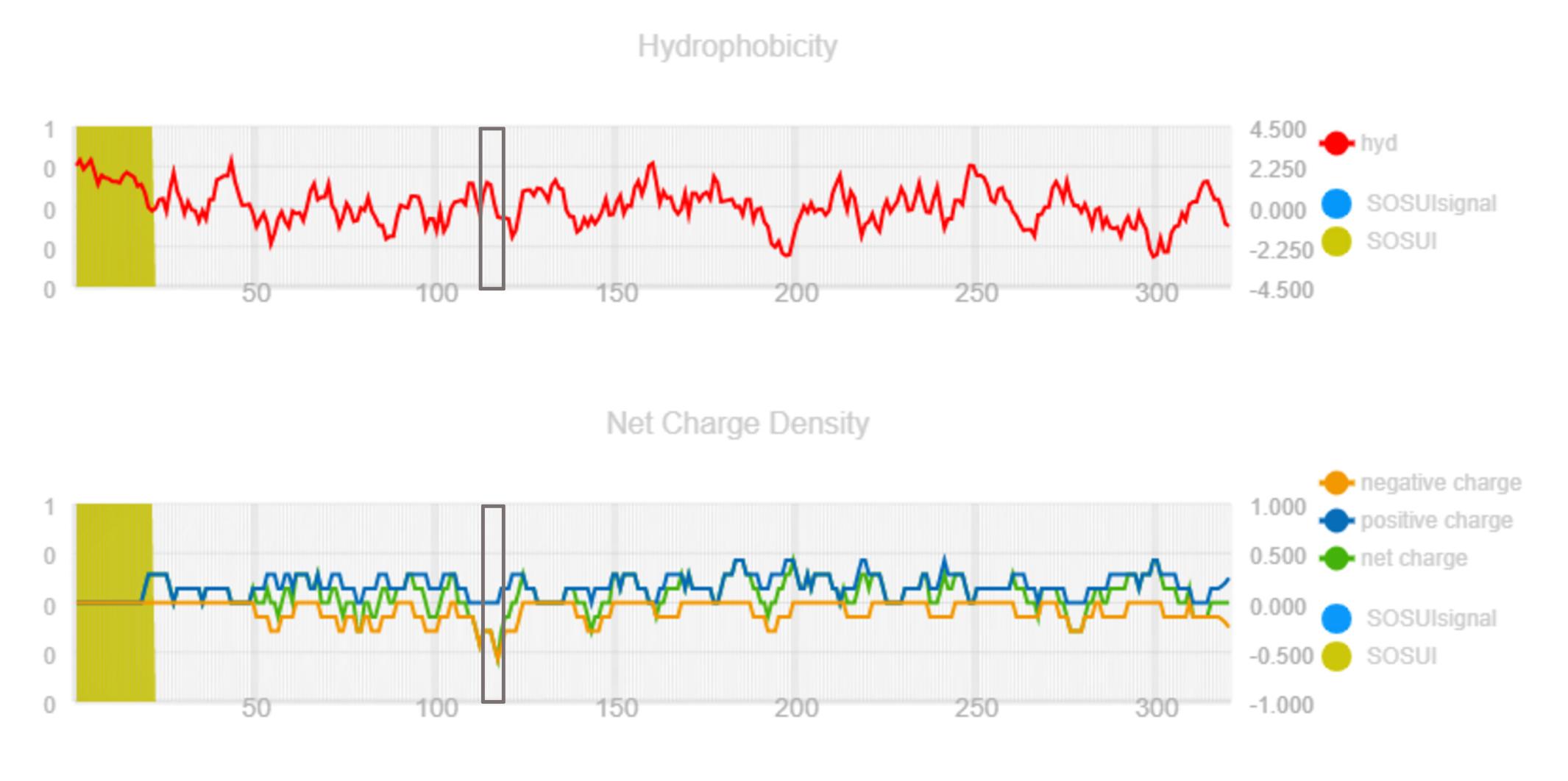


**Fig.S2 Result of SOSUI prediction to mutated RDH12*.*** The extra 5 amino acids of the mutanted RDH12 are framed by grey boxes
